# Comparison of the impact of chronic corticosteroid therapy on critical care outcomes of COVID-19 patients with and without history of chronic liver disease

**DOI:** 10.1038/s41598-021-98778-z

**Published:** 2021-09-28

**Authors:** Hammad Liaquat, Brittney Shupp, Samantha Rollins, Yecheskel Schneider, Ayaz Matin

**Affiliations:** Division of Gastroenterology, St Luke’s University Health Network (affiliated with Lewis Katz School of Medicine at Temple University), 801 Ostrum St., Suite 201, Bethlehem CampusBethlehem, PA 18015 USA

**Keywords:** Outcomes research, Viral infection, Liver, Risk factors

## Abstract

There is a paucity of studies investigating the impact of chronic corticosteroid use for coexisting conditions in patients with Coronavirus Disease 2019 (COVID-19). Additionally, the information regarding the impact of chronic liver disease (CLD) on COVID-19 outcomes is evolving. Our study aims to investigate hospitalization outcomes of patients with COVID-19 on long term corticosteroids for coexisting conditions while also seeking to compare outcomes between such patients with a history of CLD to analyze the impact on mortality. We conducted a retrospective chart review across our 10-hospital network identifying patients on chronic corticosteroids (Prednisone ≥ 5 mg daily dose or equivalent dose of another steroid, for a duration of 30 days or more) who were hospitalized with COVID-19 from March 1, 2020 to June 30, 2020. Of these patients who met inclusion criteria, patients were then divided into groups based upon their history of CLD. Primary outcomes of the study looked to investigate the hospitalization outcomes of patients with a history of CLD and comorbid conditions requiring chronic corticosteroid use. Secondary outcomes sought to further investigate risk factors for mortality in our study sample. 837 charts were reviewed. 139 patients met inclusion criteria of which 34 patients had a history of CLD. Statistical analysis demonstrated no difference in length of hospital stay but increased ICU admission rate in the CLD group (41.2% vs 23.8%). No statistically significant difference was seen in between the CLD and non-CLD groups in term of complication rates and 28-day mortality. However, chronic corticosteroids patients were found to have higher rates of ICU admission and overall 28-day and ICU mortality in comparison to patients who were not on chronic corticosteroids prior to COVID-19 hospitalization. The larger contributor to COVID-19 severity was likely chronic corticosteroid use rather than CLD and thus chronic corticosteroid use should be limited throughout the COVID-19 pandemic especially in patients with additional speculated risk factors for COVID-19 such as CLD.

## Introduction

In December 2019, the first case of Coronavirus Disease 2019 (COVID-19) was identified originating from its outbreak epicenter in Wuhan, China^1^. After its emergence, limited information was available except the information that was extrapolated from preceding coronavirus disease outbreaks including Severe Acute Respiratory Distress Syndrome (SARS) and Middle East Respiratory Distress Syndrome (MERS). This knowledge gap prompted robust research that led to a better yet limited understanding of the risk factors, appropriate treatment, and the morbidity and mortality of COVID-19. Early on, patients with comorbid conditions including diabetes, chronic lung disease, cardiovascular disease, hypertension, and cancer were labeled as being at high risk for developing severe COVID-19^2^. More recent data suggests a link between chronic liver disease (CLD), including those with and without cirrhosis, to higher rates of morbidity and mortality in COVID-19^3–5^.

Additionally, at the start of the pandemic, the World Health Organization (WHO) advised against the use of corticosteroids in the setting of COVID-19 except if required for treatment of co-existing conditions^6^. This recommendation was based upon lack of data about the use of corticosteroids in COVID-19 as well as previously documented poor outcomes in previous viral outbreaks^6,7^. Since then, studies on corticosteroids have yielded promising results and have demonstrated a potential role and benefit for corticosteroid use, specifically Dexamethasone, for short term use in severe and critically ill COVID-19 patients^8^. Unfortunately, there still remains scarcity of data and lack of retrospective or prospective studies on the outcomes of COVID-19 patients with comorbid conditions that require chronic corticosteroid use. (9) Our study aims to investigate the hospitalization outcomes of patients with a history of CLD and comorbid conditions requiring chronic corticosteroid use. Our secondary aim was to investigate risk factors for mortality in our study sample.

## Methods

### Study design

**Figure 1:**
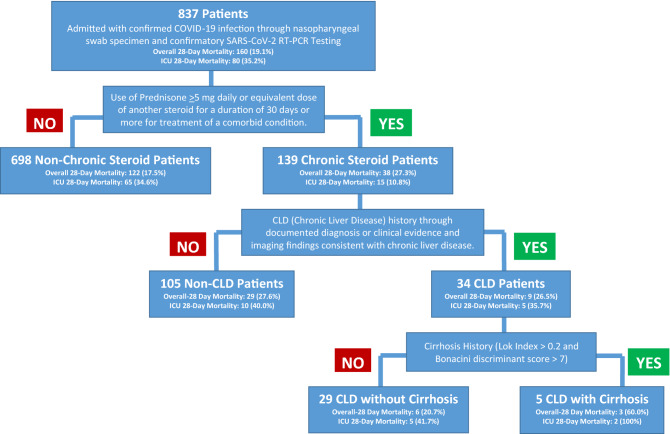
Step-wise approach utilized to obtain final patient sample of both CLD and non-CLD COVID-19 patients on chronic corticosteroids for comorbid condition.

We conducted a retrospective chart review within our 10 hospital network located in Eastern Pennsylvania. This study was approved by St. Luke’s University Health Network (SLUHN) Institutional Review Board (IRD) (IRB 00002757) who has Federal Wide Assurance (FWA 00003557) from the office for Human Research Protection (OHRP). The IRB granted a waiver of informed consent and HIPPA authorization and all methods were completed in accordance with their guidelines and regulations. We reviewed all patients who tested positive for COVID-19 with nasopharyngeal swab specimens and confirmatory SARS-CoV-2 reverse transcriptase-polymerase chain reaction (RT-PCR) testing from March 1, 2020 to June 30, 2020 (Fig. 1). Inclusion criteria included hospitalized patients who tested positive for COVID-19 and were also on long term corticosteroids (Prednisone ≥ 5 mg daily dose or equivalent dose of another steroid, for a duration of 30 days or more) for coexisting disease. Of those who met inclusion criteria, those with CLD were identified. The patients were divided based on their history of CLD (CLD group) or no history of CLD (non-CLD group). The CLD group was subsequently divided into those with or without cirrhosis for statistical analysis. Exclusion criteria included patients who tested negative for COVID-19 and COVID-19 patients on a short corticosteroid course or treated for COVID-19 outpatient.

### Data collection

A standardized data collection form was created for data retrieval from the electronic health record (EHR) system. Data was collected on baseline demographics and characteristics such as comorbid conditions, including a history of CLD. Additional collected information included patients’ classical COVID-19 symptoms (fever, cough and shortness of breath), laboratory values on day of admission, and treatment administered for COVID-19. Information regarding patients’ complications, critical care outcomes, and 28-day mortality was also collected.

### Comorbidity assessment

Patients with a documented history of CLD and cirrhosis were identified. Patients who did not carry a diagnosis but had clinical evidence (persistently elevated liver function tests and positive viral hepatitis serologies) and imaging findings consistent with liver disease (hepatic steatosis, nodularity, and echogenicity) where included in this group. For each patient with diagnosis or evidence of chronic liver disease, we calculated Lok index and Bonacini discriminant score to check if they met criteria for cirrhosis (Lok Index > 0.2 and Bonacini discriminant score > 7). For each patient, the comorbid conditions associated with the Charlson Comorbidity Index was collected to appropriately predict patients’ 10-year mortality. However, considering this study aimed to analyze clinical outcomes between CLD and non-CLD patients, a Modified Charlson Comorbidity Index (MCCI) was utilized and excluded liver disease in the risk calculation. A MCCI of 1–2 was considered a low risk predictor for 10-year morality. A MCCI of 3–4 was considered medium risk. A MCCI of greater than 4 was considered a high risk predictor.

### Statistical analysis

Both continuous and categorical variables were recorded in the data charts. Continuous variables were presented as mean ± standard deviations while categorical variables were expressed as n (%). In order to determine the statistical significance between outcomes in the CLD and non-CLD group, continuous variables were compared using the Fisher’s-exact test and Chi-Square analysis as appropriate. When comparing continuous variables, the tailored t-test was utilized to compare the means. Calculated *p*-values of < 0.05 were considered statistically significant. Additionally, odds ratio (OR) was calculated for each variable to allow for a comprehensive comparison. When data was analyzed between those with and without cirrhosis, the sample size was too small to create a comparison for statistical analysis.

### Ethics approval and consent to participate

Ethics approval was obtained from Institutional Review Board (IRB) before starting the study. No consent to participate was taken or needed (with approval from IRB) as study was retrospective in nature and based of review of patient charts.

### Consent for publication

Not applicable.

## Results

Charts of 837 patients admitted with confirmed COVID-19 infection were reviewed and 139 met inclusion criteria (Fig. 1). Overall, patients required chronic corticosteroids for reasons including asthma/COPD (n = 70; 50.3%), allergies or associated conditions (n = 27, 19.4%), rheumatic disease (n = 9; 6.5%), inflammatory bowel disease (IBD) (n = 3; 2.2%), and additional causes (n = 30; 21.6%) (Table 1). The median age of patients in the non-CLD group was 77 years (range 19–97 years) and 70 years (range 50–90 years) in the CLD patient group. Majority of the patients in both groups were elderly (65 years and above) and Caucasian. Majority of patients (69.8%) in both groups had a MCCI greater than 4 indicating that the sample included a high-risk cohort. No significant differences in demographic or comorbidities were seen between the two groups (Table 1). Duration of hospital stay also did not defer significantly between groups, but a higher proportion of patients in the CLD group (41.2% vs 23.8%) were admitted to the intensive care unit (ICU) with a difference that approached significance (*p* = 0.05).

**Table 1:** Characteristics of admitted COVID-19 patients with or without chronic liver disease (CLD) on long-term corticosteroid treatment for co-existing conditions.

Characteristics	Non-CLD (n = 105)	CLD (n = 34)	*p* value
Mean age ± S.D., years	73.9 ± 14.7	69.7 ± 10.3	0.06
Age ≥ 65 years, n (%)	78 (67.6)	21 (61.8)	0.25
Male gender, n (%)	44 (41.9)	12 (35.2)	0.49
Caucasian ethnicity, n (%)	68 (64.8)	28 (82.3)	0.06
Mean BMI ± S.D., kg/m^2^	29.6 ± 7.6	32.5 ± 9.1	0.33
Nursing home resident, n (%)	45 (42.9)	16 (47.1)	0.67
Etiology for chronic steroid treatment, n (%)			
Asthma/chronic obstructive pulmonary disease	50 (47.6)	20 (58.8)	0.25
Allergy	23 (21.9)	4 (11.8)	0.22
Rheumatoid disease	7 (6.7)	2 (5.9)	1.0
Inflammatory bowel disease	1 (0.9)	2 (5.9)	0.14
Others	24 (22.9)	6 (17.6)	0.63
Modified Charlson Comorbidity Index (MCCI)^a^, mean score ± S.D	5.6 ± 2.4	6.2 ± 2.7	0.25
MCCI^a^ 1–2	14 (13.3)	2 (5.9)	0.36
MCCI^a^ 3–4	19 (18.1)	7 (20.6)	0.85
MCCI^a^ > 4	72 (68.6)	25 (73.5)	0.79
History of myocardial Infarction, n (%)	18 (17.1)	5 (14.7)	0.73
Congestive heart failure, n (%)	37 (35.2)	9 (26.5)	0.34
Peripheral vascular disease, n (%)	7 (6.7)	0 (0.0)	0.19
History of CVA/TIA, n (%)	17 (16.2)	8 (23.5)	0.33
Dementia, n (%)	17 (16.2)	4(11.8)	0.78
COPD, n (%)	37 (35.2)	16 (47.1)	0.21
Connective tissue disease, n (%)	16 (15.2)	2 (5.8)	0.24
Diabetes/prediabetes, n (%)	45 (42.9)	20 (58.8)	0.10
Peptic ulcer disease, n (%)	4 (3.8)	1 (2.9)	1.0
Hemiplegia, n (%)	1 (1.0)	0 (0.0)	1.0
Leukemia, n (%)	1 (1.0)	1 (2.9)	0.43
Lymphoma, n (%)	1 (1.0)	0 (0.0)	1.0
HIV/AIDS, n (%)	1 (1.0)	0 (0.0)	1.0
Moderate to severe CKD, n (%)	21 (20.0)	1 (2.9)	0.01
Solid tumor ± metastasis, n (%)	16 (15.2)	9 (26.5)	0.14
Admitted to intensive care unit, n (%)	25 (23.8)	14 (41.2)	0.05
Length of hospital stay, mean ± S.D., days	9.3 ± 9.2	9.6 ± 5.6	0.47

^a^Modified Charlson Comorbidity Index: liver disease not included in calculation of score.

When comparing the classical COVID-19 presenting symptoms, there was no significant statistical difference between groups (Table 2). Admission laboratory values varied but the CLD group had statistically significant lower platelets (174.2 thousands/uL verses 224.4 thousand/uL), sodium (135.1 mmol/L vs 137.4 mmol/L) and albumin (2.8 g/dL verses 3.1 g/dL) levels and higher total bilirubin levels (0.9 mg/dL verses 0.5 mg/dL) (Table 2). The CLD patients also had higher serum AST (176.6 U/L versus 41.5 U/L), serum ALT (81.1 U/L verses 38.1 U/L), alkaline phosphatase (109.7 U/L versus 88.7 U/L), prothrombin time (24.1 s verses 15.1 s) and procalcitonin (9.5 ng/mL versus 0.9 ng/mL) levels than the non CLD group, yet these values were not statistically significant.

**Table 2:** Admission symptoms and laboratory values of admitted COVID-19 patients with or without chronic liver disease (CLD) on long-term corticosteroid treatment for co-existing conditions.

Admission symptoms and laboratory values	Non-CLD (n = 105)	CLD (n = 34)	*p* value
Admission symptoms			
Hyperthermia, n (%)	44 (43.8)	16 (47.1)	0.60
Cough, n (%)	73 (70.1)	23 (67.6)	0.83
Dyspnea, n (%)	84 (80.0)	30 (88.2)	0.31
Laboratory values on day of admission			
White blood count, mean ± S.D., thousands/uL	7.6 ± 3.7	7.14 ± 4.2	0.56
Absolute neutrophil count, mean ± S.D., thousands/uL	7.9 ± 22.4	5.25 ± 2.8	0.25
Absolute lymphocyte count, mean ± S.D., thousands/uL	1.5 ± 4.7	1.4 ± 2.8	0.85
Platelet count, mean ± S.D., thousands/uL	224.4 ± 102.6	174.2 ± 69.1	0.001
Prothrombin time, mean ± seconds	15.1 ± 4.3	24.1 ± 42.5	0.34
Activated PTT, mean ± S.D., seconds	36.0 ± 15.8	33.8 ± 11.2	0.54
D-Dimer, mean ± S.D., ug/ml	2.2 ± 4.5	1.7 ± 1.4	0.44
Creatine kinase, mean ± S.D., U/L	152.5 ± 158.8	260.5 ± 508.2	0.45
Aspartate aminotransferase, mean ± S.D., U/L	41.5 ± 32.2	176.6 ± 691.1	0.26
Alanine aminotransferase, mean ± S.D., U/L	38.1 ± 27.3	81.1 ± 206.1	0.23
Total bilirubin, mean ± S.D., mg/dL	0.5 ± 0.2	0.9 ± 1.1	0.04
Alkaline phosphatase, mean ± S.D., U/L	88.7 ± 40.6	109.7 ± 68.9	0.09
Albumin, mean ± S.D., g/dL	3.1 ± 0.5	2.8 ± 0.6	0.04
Sodium, mean ± S.D., mmol/L	137.4 ± 5.8	135.1 ± 4.9	0.02
Blood urea nitrogen, mean ± S.D., mg/dL	28.5 ± 20.83	27.5 ± 16.8	0.78
Creatinine, mean ± S.D., mg/dL	1.6 ± 1.6	1.6 ± 1.4	0.86
Lactic acid, mean ± S.D., mmol/L	1.7 ± 0.9	2.0 ± 1.5	0.27
Procalcitonin, mean ± S.D., ng/mL	0.9 ± 2.7	9.5 ± 42.1	0.34

When looking at overall hospitalization outcomes, a statistically significant higher percentage of patients in the CLD group required additional treatment with high dose steroids (67.6% verses 45.7%) (Table 3). A higher proportion of CLD patients were also found to have required vasopressor support and supplemental oxygen (including nasal canula (NC), mid flow NC, and high flow NC) and/or mechanical ventilation. Antibiotics and hydroxychloroquine was also given to larger fraction of CLD patients than those without CLD. The hospital course of a higher proportion of patients with CLD patients was complicated by the development of secondary infection (including both respiratory and urologic infections), respiratory distress syndrome (ARDS), arrhythmia, and acute kidney injury (AKI) but the difference between patient's without CLD was not significant.

**Table 3:** Hospitalization and critical care outcomes of admitted COVID-19 patients with or without chronic liver disease (CLD).

Characteristics	Non-CLD (n = 105)	CLD (n = 34)	*p* value
**Patients requiring hospitalization**	105	34	
Treatment during hospital stay			
High dose steroids, n (%)	48 (45.7)	23 (67.6)	0.02
Vasopressors, n (%)	16 (15.2)	8 (23.5)	0.26
Plaquenil, n (%)	79 (75.2)	29 (85.3)	0.22
Antivirals, n (%)	8 (7.6)	1 (2.9)	0.45
Antibiotics, n (%)	80 (76.2)	29 (85.3)	0.26
CRRT*, n (%)	2 (1.9)	0 (0.0)	1.0
Oxygen requirement above baseline, n (%)	70 (66.6)	24 (70.5)	0.67
Mechanical Ventilation, n (%)	11 (10.5)	7 (20.6)	0.74
Complications during hospital stay			
Secondary infection, n (%)	45 (42.9)	17 (50.0)	0.46
Arrhythmias, n(%)	11 (10.5)	8 (23.5)	0.06
Acute cardiac injury, n(%)	11 (10.5)	2 (5.9)	0.73
TIA/CVA, n (%)	1 (1.0)	0 (0.0)	1.0
ARDS, n (%)	41 (39.0)	19 (55.9)	0.08
Acute kidney injury, n (%)	41 (39.0)	16 (47.1)	0.41
28 day mortality, n (%)	29 (27.6)	9 (26.5)	0.89
**ICU admissions**	25	14	
Treatment during ICU stay			
High dose steroids, n (%)	19 (76.0)	13 (92.9)	0.38
Vasopressors, n (%)	11 (44.0)	7 (50.0)	0.71
Plaquenil, n (%)	23 (92.0)	11 (78.6)	0.50
Antivirals, n (%)	7 (28.0)	1 (7.1)	0.21
Antibiotics, n (%)	23 (92.0)	14 (100.0)	0.52
CRRT*, n (%)	2 (8.0)	0 (0.0)	0.52
Oxygen requirement above baseline, n (%)	23 (92.0)	13 (92.8)	0.92
Mechanical ventilation, n (%)	11 (44.0)	7 (50.0)	0.71
Complications during ICU stay			
Secondary infection, n (%)	16 (64.0)	8 (57.1)	0.67
Acute cardiac injury, n(%)	2 (8.0)	2 (5.9)	0.60
Arrhythmias, n (%)	5 (20.0)	6 (42.9)	0.13
TIA/CVA**, n (%)	0 (0.0)	0 (0.0)	NA
ARDS***, n (%)	18 (72.0)	12 (85.7)	0.44
Acute kidney injury, n (%)	11 (44.0)	8 (57.1)	0.43
28 day mortality, n (%)	10 (40.0)	5 (35.7)	0.79
**Patients on mechanical ventilation in ICU**	11	7	
Duration between ICU**** admission and steroid initiation, mean ± S.D., days	2.0 ± 3.2	1.7 ± 1.9	0.74
Duration between mechanical ventilation and steroid initiation, mean ± S.D., days	4.1 ± 4.2	2.3 ± 2.1	0.31
PaO2/FiO2*****, mean ± S.D	200.1 ± 107.4	128.4 ± 75.3	0.14
Mild, n (%)	3 (27.3)	1 (14.3)	0.04
Moderate, n (%)	7 (63.6)	6 (85.7)	0.59
Severe, n (%)	1 (9.1)	0 (0.0)	1.0
Duration of mechanical ventilation, mean ± S.D., days	15.1 ± 8.8	4.9 ± 4.1	0.003
Length of ICU stay, mean ± S.D., days	11.2 ± 10.7	5.5 ± 4.2	0.02
28 day mortality, n (%)	5 (45.5)	3 (42.9)	1.0

CRRT*, continuous renal replacement therapy; TIA/CVA**, transient ischemic attack/cerebrovascular accident; ARDS***, acute respiratory distress syndrome; ICU****, intensive care unit; PaO2/FiO2*****, ratio of arterial oxygen partial pressure (PaO2) to fractional inspired oxygen (FiO2).

When critical care management in patient requiring ICU admission (Table 3), a higher fraction of patients in CLD group required additional corticosteroid treatment, vasopressors, antibiotics and mechanical ventilatory support but the difference was without statistical significance. Development of secondary infection, acute cardiac injury was more prevalent in patients without CLD while arrhythmias, ARDS and AKI developed more frequently in the CLD group. Although all differences did not reach statistical significance. Duration between ICU admission (*p* = 0.74), mechanical ventilation (*p* = 0.31) and initiation of high-dose steroid treatment was shorter in patients with CLD yet not significant (Table 3). However, patients in the non-CLD group remained on mechanical ventilation for a longer duration of time (*p* = 0.003) and had longer length of ICU stay (*p* = 0.02) both of which were significant. Hospitalization data for patients with a diagnosis of cirrhosis was also analyzed and compared to patients without cirrhosis within the CLD group but due to the significantly low sample of cirrhotic patients (n = 5) no definitive conclusions were drawn from the analysis (Table 4).

**Table 4:** Management and outcomes of hospitalization and critical care in admitted COVID-19 patients with or without cirrhosis.

Characteristics	CLD with no history of cirrhosis (n = 29)	History of cirrhosis (n = 5)
**Patients requiring hospitalization**	29	5
Treatment during hospital stay		
High dose steroids, n (%)	19 (65.5)	4 (80.0)
Vasopressors, n (%)	6 (20.7)	2 (40.0)
Plaquenil, n (%)	25(86.2)	4 (80.0)
Antivirals, n (%)	1 (3.4)	0 (0.0)
Antibiotics, n (%)	25 (86.2)	4 (80.0)
CRRT*, n (%)	0 (0.0)	0 (0.0)
Mechanical Ventilation, n (%)	6 (21)	1 (20.0)
Complications during hospital stay		
Secondary Infection, n (%)	14 (48.3)	3 (60.0)
Acute Cardiac Injury, n(%)	2 (6.9)	0 (0.0)
TIA/CVA**, n (%)	0 (0.0)	0 (0.0)
Arrhythmias, n(%)	7 (24.1)	1 (20.0)
ARDS***, n (%)	16 (55.2)	3 (60.0)
Acute Kidney Injury, n (%)	13 (44.8)	3 (60.0)
28 Day Mortality overall, n (%)	6 (20.7)	3 (60.0)
**Intensive Care Unit admissions**	12	2
Treatment during stay in ICU****		
High dose steroids, n (%)	12 (100.0)	1 (50.0)
Vasopressors, n (%)	6 (50.0)	1 (50.0)
Plaquenil, n (%)	10 (83.3)	1 (50.0)
Antivirals, n (%)	1 (8.3)	0 (0.0)
Antibiotics, n (%)	12 (100.0)	2 (100.0)
CRRT*, n (%)	0 (0.0)	0 (0.0)
Mechanical ventilation, n (%)	6 (50.0)	1 (50.0)
Complications during stay in ICU****		
Secondary Infection, n (%)	6 (50.0)	2 (100.0)
Acute Cardiac Injury, n(%)	2 (16.7)	0 (0.0)
TIA/CVA**, n (%)	0 (0.0)	0 (0.0)
Arrhythmias, n (%)	6 (50.0)	0 (0.0)
ARDS***, n (%)	10 (83.3)	2 (100.0)
Acute Kidney Injury, n (%)	6 (50.0)	2 (100.0)
Patients on mechanical ventilation in ICU****	6	1
Duration between Intensive Care Unit admission and steroid initiation, mean ± S.D., days	1.8 ± 2.1	3
Duration between mechanical ventilation and steroid initiation, mean ± S.D., days	2.3 ± 1.9	0
PaO2/FiO2****, mean ± S.D	117.4 ± 70.4	183.1
Mild, n (%)	1 (16.6)	0 (0.0)
Moderate, n (%)	5 (83.3)	1 (100.0)
Severe, n (%)	0 (0.0)	0 (0.0)
Duration of mechanical ventilation, mean ± S.D., days	5.33 ± 3.9	2
Length of Intensive Care Unit stay, mean ± S.D., days	6.3 ± 4.3	1
28 day mortality n, (%)	3 (50.0)	1 (100.0)

CRRT*, continuous renal replacement therapy; TIA/CVA**, transient ischemic attack/cerebrovascular accident; ARDS ***, acute respiratory distress syndrome; ICU****, intensive care unit; PaO2/FiO2*****, ratio of arterial oxygen partial pressure (PaO2) to fractional inspired oxygen (FiO2).

There was no significant impact of history of CLD upon mortality (n = 139, *p* = 0.89) (Table 5). Completion of a risk factor analysis demonstrated that within the non-CLD group, elderly (aged ≥ 65 years and older) patients (OR 6.2, 95%CI 1.4–28.3, *p* = 0.01) and nursing home residents (OR 3.7, 95%CI 1.5–8.9, *p* = 0.005) were significant risk factors associated with mortality. Additionally, the requirement of oxygen support and/or vasopressors and the development of arrhythmias, ARDS or AKI were major risk factors associated with mortality amongst patients without CLD. Amongst the CLD cohort, only higher comorbidity reflected by a MCCI > 4 (OR 13.2, 95% CI 0.7–251.0, *p* = 0.03), development of secondary infection (OR 14.2, 95% CI 1.5–132.7, *p* = 0.02) and AKI (OR 6.2, 95% CI 1.1–36.7, *p* = 0.05) had a significant association with patient death during hospitalization.

**Table 5:** Predictors of mortality in patients with or without history of CLD.

Characteristics	Non-CLD, (n = 105)				CLD, (n = 34)			
	Died (29)	Alive (76)	OR (95% CI)	*p* value	Died (9)	Alive (25)	OR (95% CI)	*p* value
Age ≥ 65 years, n (%)	27 (93.1)	52 (68.4)	6.2 (1.4–28.3)	0.01	8 (88.9)	13 (52.0)	7.4 (0.8–68.1)	0.11
Male gender, n (%)	9 (31.0)	35 (46.1)	0.5 (0.2–1.3)	0.17	4 (44.4)	8 (32.0)	1.7 (0.4–8.1)	0.69
Caucasian ethnicity, n (%)	23 (79.3)	45 (59.2)	2.6 (0.9 -7.2)	0.06	7 (77.8)	21 (84.0)	0.5 (0.1–3.6)	0.60
BMI ≥ 30 kg/m^2^, n (%)	11 (37.9)	32 (42.1)	0.8 (0.3–2.0)	0.70	3 (33.3)	13 (52.0)	0.5 (0.1–2.3)	0.45
Nursing home resident, n (%)	19 (65.5)	26 (34.2)	3.7(1.5–8.9)	0.005	6 (66.7)	10 (40.0)	3.0 (0.6–14.9)	0.25
MCCI* 1–2, n (%)	2 (6.9)	12 (15.8)	0.4 (0.08–1.9)	0.34	0 (0.0)	2 (8.0)	0.5 (0.02–11.3)	1.00
MCCI* 3–4, n (%)	3 (10.3)	16 (21.1	0.4 (0.1–1.6)	0.26	0 (0.0)	7 (28.0)	0.1 (0.01–2.5)	0.15
MCCI* > 4, n (%)	24 (82.8)	48 (63.2)	2.8 (0.9–8.2)	0.06	9 (100.0)	16 (64.0)	13.2 (0.7–251.0)	0.03
History of myocardial infarction, n (%)	8 (27.6)	10 (13.2)	2.5 (0.9–7.2)	0.09	1 (11.1)	4 (16.0)	0.7 (0.1–6.8)	1.00
Congestive heart failure, n (%)	15 (51.7)	22 (29.0)	2.6 (1.1 -6.3)	0.03	4 (44.4)	5 (20.0)	3.2 (0.6–16.5)	0.20
Peripheral vascular disease, n (%)	4 (13.8)	3 (3.9)	3.9 (0.8–18.6)	0.08	0 (0.0)	0 (0.0)	NA	NA
History of TIA/CVA**, n (%)	5 (17.2)	12 (15.8)	1.1 (0.4–3.5)	0.86	4 (44.4)	4 (16.0)	4.2 (0.8–22.9)	0.16
Dementia, n (%)	9 (31.0)	8 (10.5)	3.8 (1.3–11.2)	0.01	0 (0.0)	4 (16.0)	0.3 (0.01–5.2)	0.55
COPD***, n (%)	11 (37.9)	26 (34.2)	1.2 (0.5 -2.9)	0.69	3 (33.3)	13 (52.0)	0.5 ((0.1–2.3)	0.45
Connective tissue disease, n (%)	5 (17.2)	11 (14.5)	1.2 (0.4–3.9)	0.72	0 (0.0)	2 (8.0)	0.5 (0.02–11.3)	1.00
Diabetes/prediabetes, n (%)	10 (34.5)	35 (46.1)	0.6 (0.3–1.5)	0.29	6 (66.7)	14 (56.0)	1.6 (0.3–7.7)	0.70
Peptic ulcer disease, n (%)	0 (0.0)	4 (5.3))	0.3 (0.01–5.2)	0.57	0 (0.0)	1 (4.0)	0.9 (0.03–23.0)	1.00
Hemiplegia, n (%)	0 (0.0)	1 (1.3)	0.9 (0.03–21.5)	1.00	0 (0.0)	0 (0.0)	NA	NA
Leukemia, n (%)	1 (3.4)	0 (0.0)	8.1 (0.3–203.1)	0.28	1 (11.1)	0 (0.0)	9.0 (0.3–242.4)	0.26
Lymphoma, n (%)	0 (0.0)	1 (1.3)	0.9 (0.03–21.5)	1.00	0 (0.0)	0 (0.0)	NA	NA
HIV/AIDS****, n (%)	0 (0.0)	1 (1.3)	0.9 (0.03–21.5)	1.00	0 (0.0)	0 (0.0)	NA	NA
Moderate to severe CKD, n (%)	10 (34.5)	11 (14.5)	3.1 (1.1 -8.4)	0.03	1 (11.1)	0 (0.0)	9.0 (0.3–242.4)	0.26
Solid tumor ± metastasis, n (%)	9 (31.0)	7 (9.2)	4.4 (1.5–13.4)	0.01	3 (33.3)	6 (24.0)	6.8 (1.8–26.0)	0.67
Admission symptoms								
Hyperthermia, n (%)	11 (37.9)	33 (43.4)	0.8 (0.3–1.9)	0.61	4 (44.4)	12 (48.0)	0.9 (0.2–4.0)	1.00
Cough, n (%)	22 (75.9)	51 (67.1)	1.5 (0.6–4.1)	0.39	5 (55.6)	18 (72.0)	0.5 (0.1–2.4)	0.43
Dyspnea, n (%)	29 (100.0)	55 (72.4)	22.9 (1.3–390.1)	0.0007	9 (100.0)	21 (84.0)	4.0 (0.2–81.5)	0.55
Hospitalization treatment								
High dose steroids, n (%)	17 (58.6)	31 (40.8)	2.1 (0.7–4.9)	0.10	6 (66.7)	17 (68.0)	9.5 (2.2–41.9)	1.00
Vasopressors, n (%)	8 (27.6)	8 (10.5)	3.2 (1.1–9.7)	0.04	3 (33.3)	5 (20.0)	2.0 (0.4–10.9)	0.65
Plaquenil, n (%)	23(79.3)	56 (73.7)	1.4 (0.5–3.8)	0.55	8 (88.9)	21 (84.0)	1.5 (0.1–15.8)	1.00
Antivirals, n (%)	3 (10.3)	5 (6.6)	1.6 (0.4–7.3)	0.68	1 (11.1)	0 (0.0)	9.0 (0.3–242.4)	0.26
Antibiotics, n (%)	26 (89.7)	54 (71.1)	3.5 (1.0–12.9)	0.07	9 (100.0)	20 (80.0)	5.1 (0.3 -101.9)	0.29
CRRT*****, n (%)	1 (3.4)	1 (1.3)	2.7 (0.2–44.3)	0.48	0 (0.0)	0 (0.0)	NA	NA
Oxygen requirement above baseline, n (%)	26 (89.7)	44 (57.9)	6.3 (1.8–22.6)	0.002	7 (77.8)	17 (68.0)	1.6 (0.3–9.8)	0.69
Mechanical ventilation, n (%)	5 (17.2)	6 (7.9)	2.4 (0.7–8.7)	0.17	3 (33.3)	4 (16.0)	2.6 (0.5–15.1)	0.35
Hospitalization complications:								
Secondary infection, n (%)	16 (55.2)	29 (38.2)	2.0 (0.8–4.7)	0.12	8 (88.9)	9 (36.0)	14.2 (1.5–132.7)	0.02
Acute cardiac injury, n(%)	4 (13.8)	7 (9.2)	1.6 (0.4–5.9)	0.49	0 (0.0)	2 (8.0)	0.5 (0.02–11.3)	1.0
Arrhythmias, n(%)	7 (24.1)	4 (5.2)	5.7 (1.5–21.4)	0.009	3 (33.3)	5 (20.0)	2.0 (0.4–10.9)	0.65
TIA/CVA**, n (%)	0 (0.0)	1 (1.3)	0.9 (0.03–21.5)	1.00	0 (0.0)	0 (0.0)	NA	NA
ARDS***, n (%)	21 (72.4)	20 (26.3)	7.4 (2.8–19.2)	< 0.0001	7 (77.8)	12 (48.0)	3.8 (0.7–22.0	0.24
Acute kidney injury, n (%)	19 (65.5)	22 (28.9)	4.7 (1.9–11.6)	0.0009	7 (77.8)	9 (36.0)	6.2 (1.1–36.7)	0.05
Intensive Care unit admissions	10	15			**5**	**9**		
Intensive Care unit treatment								
High dose steroids, (%)	8 (80.0)	11 (73.3)	1.5 (0.2 -10.0)	1.00	4 (80.0)	9 (100.0)	0.2 (0.01–4.7)	0.36
Vasopressors, n (%)	6 (60.0)	5 (33.3)	3.3 (0.6 -17.2)	0.23	2 (40.0)	5 (55.6)	0.5 (0.1–4.9)	1.00
Plaquenil, n (%)	10 (100.0)	13 (86.7)	3.9 (0.2–90.0)	0.50	4 (80.0)	7 (77.8)	1.1 (0.1–16.9)	1.00
Antivirals, n (%)	3 (30.0)	4 (26.7)	1.2 (0.2–6.9)	1.00	1 (20.0)	0 (0.0)	6.3 (0.2–188.2)	0.36
Antibiotics, n (%)	10 (100.0)	13 (86.7)	3.9 (0.2–90.0)	0.50	5 (100.0)	9 (100.0)	0.6 (0.01–33.5)	NA
CRRT*****, n (%)	1 (10.0)	1 (6.7)	1.6 (0.1–28.1)	1.00	0 (0.0)	0 (0.0)	NA	NA
Oxygen requirement above baseline, n (%)	10 (100.0)	13 9 (86.7)	3.9 (0.2–90.0)	0.50	4 (80.0)	9 (100.0)	0.2 (0.01–4.7)	0.36
Mechanical Ventilation, n (%)	5 (50.0)	6 (40.0)	1.5 (0.3–7.5)	0.62	3 (60.0)	4 (44.4)	1.9 (0.2–17.3)	1.00
Intensive care unit admissions	10	15			**5**	**9**		
Complication intensive care unit								
Secondary infection, n (%)	7 (70.0)	9 (60.0)	1.6 (0.3–8.5)	0.69	5 (100.0)	3 (33.3)	20.4 (0.9–488.0)	0.03
Acute cardiac injury, n(%)	7 (70.0)	4 (36.7)	6.4 (1.1–37.7)	0.04	0 (0.0)	2 (22.2)	0.3 (0.01–6.9)	0.50
Arrhythmias, n(%)	4 (40.0)	1 (6.7)	9.3 (0.9–102.0)	0.12	2 (40.0)	4 (44.4)	0.8 (0.1–7.7)	1.00
TIA/CVA**, n (%)	0 (0.0)	0 (0.0)	NA	NA	0 (0.0)	0 (0.0)	NA	NA
ARDS***, n (%)	9 (90.0)	9 (60.0)	6.0 (0.6–60.4)	0.18	4 (80.0)	8 (88.9)	1.0 (0.1–14.6)	1.00
Acute kidney injury, n (%)	7 (70.0)	4 (26.7)	6.4 (1.1–37.7)	0.04	4 (80.0)	4 (44.4)	5.0 (0.4–64.4)	0.30

MCCI*, Modified Charlson Comorbidity Index (liver disease not included in calculation of score); TIA/CVA**, transient ischemic attack/cerebrovascular accident; COPD***, chronic obstructive pulmonary disease; HIV/AIDS****, human immunodeficiency virus/acquired immunodeficiency syndrome; CRRT *****, continuous renal replacement therapy.

A majority of patients who died in both groups (58.6% non-CLD vs 66.7% CLD) received high dose steroids (Table 5). In comparison, a minority of the surviving non-CLD patients (40.8%) received the same treatment. Amongst the 48 non-CLD patients who received high dose steroids after admission, 85.5% (41/48) had oxygen requirements above baseline of which 36.5% (15/48) died. Additionally, 18.8% (9/48) required mechanical ventilation of which 44.4% (4/48) died.

Data was also collected comparing ICU admission rates and 28-day mortality to patients on chronic corticosteroids versus those without. Patients who were not taking chronic corticosteroids had a 26.9% ICU admission rate along with a 34.6% ICU mortality rate. Chronic corticosteroid steroid patients had a similar 28.1% ICU admission rate but a lower 10.8% ICU mortality rate. However, the overall 28-day mortality rate was higher in those who on chronic corticosteroids (26.5%) than those who were not (17.5%).

## Discussion

Our single network, retrospective study examined the association between COVID-19 and chronic corticosteroid use, specifically in patients with CLD. Our cohort included 139 patients with 34 patients in the CLD group and 105 in the non-CLD group. Both groups included a high-risk cohort based upon MCCI and patients had similar demographics and comorbidities. Statistical analysis demonstrated no difference in length of hospital stay but increased ICU admission rate in the CLD group. Overall throughout hospital admission, a significantly larger proportion of the CLD group required additional high dose corticosteroids. However, there were no statistically significant differences in between the CLD and non-CLD groups in terms of complication rates and overall 28-day mortality. At the completion of our study, the significant predictors of mortality in the CLD group included: an MCCI score of greater than four and development of a secondary infection in the setting of COVID-19.

Our study is unique in that it specifically looked to identify the impact of chronic corticosteroid use in the era of the COVID-19 pandemic. Although the RECOVERY Trial and additional randomized control studies have investigated the use of dexamethasone and other corticosteroids for COVID-19 treatment, studies investigating the impact of chronic corticosteroid usage for coexisting conditions in COVID-19 patients remain limited^8^. In this study, all patients who met inclusion criteria were on chronic corticosteroid therapy prior to their COVID-19 diagnosis. However, 28-day mortality and ICU admission rates were also collected for the remaining patients who were excluded from the trial. Results found that the overall 28-day mortality rate was much higher for patients on chronic corticosteroids (27.3%) in comparison to those who were not (excluded patients; 17.5%) Data further differed when comparing ICU admission rates and mortality. The rate of ICU admission was comparable between groups (28.1% vs 26.9%) yet rate of ICU mortality differed significantly (10.8 vs 34.6%). Given the higher overall 28-day mortality rate of the chronic corticosteroid group, this disparity can be likely contributed to the increased number of chronic corticosteroid group patients who decompensated and changed code-status before ICU level of care could be obtained.

Unfortunately, avoidance of corticosteroids in many cases is often not an option making further investigation of the impact of their use imperative. In the realm of gastrointestinal diseases, corticosteroids are frequently indicated in cases of inflammatory bowel disease (IBD) and autoimmune conditions often as the first line of recommended treatment^9^. One of the larger studies conducted was the Surveillance Epidemiology of Coronavirus Under Research Exclusion (SECURE-IBD) analysis which looked at numerous variables including the association between immunosuppressant treatment and the clinical course of COVID-19 in IBD patients^10^. This study reported a total of 525 cases from 33 different countries of which 37 patients (7.0%) received oral/parenteral corticosteroids. Ultimately, patients taking systemic corticosteroids had higher rates of ICU admission (16%), ventilator use (14%), and/or death (11%). Overall, age (> 70 years of age), increased number of comorbidities, and corticosteroid use were the only variables associated with poorer outcomes. In comparison to the 105 non-CLD patients on chronic corticosteroids in our study with an average age of 73.9 years, 25 (23.8%) required ICU admission, 11 (10.5%) required ventilator use, and 29 (27.6%) died. Therefore, the results of the SECURE-IBD analysis alongside our study broadly looking at corticosteroid use, suggest poor outcomes related to chronic corticosteroids and therefore advise that they not be utilized unless clinically necessary.

The results of our study are two-fold in that they further look to identify the effects of corticosteroids specifically in CLD patients. Studies have been completed to help identify whether or not liver disease should be considered a risk factor for COVID-19. Currently, the Center for Disease Control recognizes that CLD might increase patient’s risk for severe COVID-19. One large multicenter study in the United States was published investigating the predictors of outcomes of COVID-19 in patients with chronic liver disease^11^. This study is the largest cohort to date involving 867 CLD patients including those with alcoholic liver disease (ALD), decompensated cirrhosis, and hepatocellular carcinoma (HCC). Their data showed that 48.9% of patients required oxygen supplementation (vs 70.5% in our study), 23% were admitted to ICU (vs 41.2% in our sample), and 14.0% was the all-cause mortality (vs 26.5% in our sample). This study ultimately concluded that the baseline ALD, decompensated cirrhosis, and HCC were all liver specific predictors of all cause morality while increased age, diabetes, hypertension, COPD, and smoking history also were significant contributing factors.

Outside these completed studies, it can further be hypothesized that patients with chronic liver disease that require corticosteroid treatment such those with liver transplants or autoimmune hepatitis could theoretically be at even higher risk of severe COVID-19 infection. Only small case series have been conducted on this subset of patients and data remains limited^9^. The European Liver and Intestine Transplantation Association (ELITA) established a registry to monitor liver transplant patients with COVID-19^12,13^. Immunosuppression with corticosteroids was not explicitly looked at but their research demonstrated that older age and patient’s with more distant liver transplants were at increased mortality risk. Our study showed comparable results when comparing the CLD vs non-CLD group patients all of whom were on chronic corticosteroids. The differences were seen when comparing the patients on chronic corticosteroids verses those who were not. Therefor in our study, the larger contributor to COVID-19 severity was likely chronic corticosteroid use rather than evidence of chronic liver disease. Given that liver disease is a speculated risk factor for COVID-19, special attention and care should provide to these patients and especially those who also require chronic corticosteroid use.

However, our study is not without fault and does possess the limitations of any retrospective analysis. First and foremost, identifying patient’s comorbidities were determined based off individual chart review and required the accuracy of appropriate documentation. Additionally, a majority of our sample included elderly, nursing home patients. Many of these patients presented with advanced symptoms or illness and were managed by treatment protocols that evolved as our understanding of COVID-19 expanded. Therefore, some patients were treated with additional high dose steroids while patients earlier in the course of the pandemic were not. Lastly, in regards to the CLD and cirrhosis group patients, our sample size was small given the fact that our study was limited to only include patients on chronic corticosteroids. This small sample size made it difficult to achieve statistical significance. Despite these drawbacks, the strengths of our study lie in that it is the largest cohort to date that broadly looks at chronic corticosteroid use for coexisting conditions. Additionally, the study is the first of its kind in that we examined the subset of patients with CLD disease and the impact of chronic corticosteroids on these patients. Being that this is a pilot, our study requires a larger and more robust study to be completed to substantiate our claims and confirm causality between long term corticosteroids and adverse COVID-19 outcomes during the ongoing pandemic.

## Conclusion

In summary, chronic use of corticosteroids may place patients at increased risk of adverse outcomes of COVID-19. Providers should ensure that corticosteroids are used cautiously and at the lowest effective dose when absolutely necessary during the ongoing pandemic. Patients requiring chronic steroid use, especially CLD patients and in particular those who require corticosteroid use, should be prioritized educated on the importance vaccination due to their increased risk of ICU admission, complications, and mortality in the setting of COVID-19 infection.

## Data Availability

The datasets used and/or analyzed during the current study are available from the corresponding author on reasonable request.
